# Metacestodes of Elasmobranch Tapeworms in *Octopus vulgaris* (Mollusca, Cephalopoda) from Central Mediterranean—SEM and Molecular Data

**DOI:** 10.3390/ani10112038

**Published:** 2020-11-04

**Authors:** Perla Tedesco, Monica Caffara, Andrea Gustinelli, Graziano Fiorito, Maria Letizia Fioravanti

**Affiliations:** 1 Department of Veterinary Medical Sciences, Alma Mater Studiorum University of Bologna, via Tolara di Sopra 50, 40064 Ozzano Emilia (B.O.), Italy; monica.caffara@unibo.it (M.C.); andrea.gustinelli2@unibo.it (A.G.); marialeti.fioravanti@unibo.it (M.L.F.); 2 Department of Biology and Evolution of Marine Organisms, Stazione Zoologica Anton Dohrn, Villa Comunale, 80121 Napoli, Italy; graziano.fiorito@szn.it

**Keywords:** octopus, cephalopod, parasite, cestode, life cycle, SEM, 28S rDNA, *Acanthobothrium*, *Anthobothrium*, *Nybelinia*

## Abstract

**Simple Summary:**

Information on the role of cephalopods in the life cycle of elasmobranch tapeworms and other parasites transmitted through the food web is limited. Such knowledge is useful to gain further understanding of the biology and ecology of this group of mollusks and would inform a correct management of wild cephalopod stocks for conservation and fishery purposes. In the present work, we aimed to characterize tapeworms infecting the common octopus *Octopus vulgaris*, one of the most widely distributed and commercially important cephalopod species, with morphological and molecular methods. Our results suggest a possible important role of *O. vulgaris* in the transmission of elasmobranch tapeworms and add valuable information on the host-range and distribution of the tapeworms identified.

**Abstract:**

Cephalopods are intermediate/paratenic hosts in the life cycle of elasmobranch tapeworms, nevertheless most records of infection in this group of mollusks are outdated and fragmentary. The present work aimed to investigate the cestode fauna of the common octopus *Octopus vulgaris* from the Tyrrhenian Sea (Central Mediterranean). The parasitic stages were characterized by light and Scanning Electron Microscopy (SEM) and sequencing of 28S rDNA. Three cestode taxa were identified to the genus level: the onchoproteocephalidean *Acanthobothrium* sp. (prevalence 28%), the “tetraphyllidean” *Anthobothrium* sp. (prevalence 13%) and the trypanorhynch *Nybelinia * sp. (prevalence 3%). The remarkable prevalence observed for gastrointestinal cestodes highlight a possible important role of *O. vulgaris* in the transmission of elasmobranch tapeworms, particularly Onchoproteocephalideans. Furthermore, the present work provides, for the first time, detailed morphological (SEM) and molecular support to confirm the occurrence of *Anthobothrium* sp. in cephalopod hosts. In order to gain higher taxonomic resolution for the identified taxa, we stress the need to collect further morphological and molecular data of adult cestodes infecting their elasmobranch definitive hosts.

## 1. Introduction

Cephalopods can host a diversity of larval cestodes, which mainly infect their digestive tract [1,2,3,4] but can also occur free in the mantle cavity or encysted in the mantle musculature [5,6]. Knowledge of the role of cephalopods in the life cycle of elasmobranch tapeworms is limited, since information on the biology and ecology of this group of parasites are still scarce [7,8]. Either eggs or free-swimming coracidia (e.g., in some trypanorhynch species) are ingested by first intermediate hosts (invertebrates) in which procercoids develop; metacestodes are found in cephalopods and other invertebrates, in teleosts and in cetaceans as second intermediate/paratenic hosts and are trophically transmitted to the definitive host represented by elasmobranchs (skates, rays and sharks) in which they develop into adults [9,10].

The numerous reports of infection by elasmobranch tapeworms [11] suggest an important role of cephalopods as intermediate/paratenic hosts in the life cycle of these parasites, nevertheless most of the available information is decades/centuries-old and fragmentary [12]. Particularly, oceanic squids are important hosts in the life cycle of Trypanorhynchs (*Nybelinia* spp., *Tentacularia coryphaenae*) and Phyllobothriids (*Phyllobothrium* spp., *Pelichnibothrium* spp.) [13,14], while for octopods, records of infection with cestode parasites are scarce [11].

Members of the genus *Octopus* Cuvier, 1798 host cestodes belonging to the orders Trypanorhyncha, Phyllobothriidea, Onchoproteocephalidea and to the non-monophyletic order “Tetraphyllidea” [1,15,16,17,18,19,20]. With respect to the Mediterranean Sea, the phyllobothridean *Phyllobothrium* sp. and the trypanorhynchs *Nybelinia lingualis* (Cuvier, 1817) Dollfus, 1933 and *Tetrarhynchus corollatus* von Siebold, 1850 (synonymized with *N. lingualis*, see Reference [10]) have been reported from the common octopus *Octopus vulgaris* Cuvier, 1797, while the “tetraphyllidean” *Scolex pleuronectis* Müller, 1788 has been reported from both *O. vulgaris* and the spider octopus *O. salutii* Vérany, 1836.

The present work aimed to investigate cestodes infecting *O. vulgaris* from Tyrrhenian Sea (Southern Italy, Central Mediterranean) through morphological analysis by light microscopy (Leica DMLS, Leica Microsystem, Milan, Italy) and Scanning Electron Microscopy (SEM) (JEOL, Basiglio, Italy) and through molecular analysis of partial 28S rDNA.

## 2. Materials and Methods

### 2.1. Sample Collection and Preservation

Fresh dead adult specimens of *O. vulgaris* (*n* = 32) were obtained from different areas in the Gulf of Naples (Tyrrhenian Sea, Mediterranean Sea) from local fishermen in the period March 2015–February 2016. Octopuses were dissected and examined for the presence of helminths in the body cavity and in organs of the digestive tract. Collected worms were washed in saline (8‰) and stored in 70% ethanol for further analyses. For permanent mounts, fixed specimens were stained with Semichon’s acetocarmine, destained in 1% acid ethanol, dehydrated in a graded ethanol series and mounted in Canada balsam. Line drawings were made with the help of a camera lucida and all measurements were taken with the imaging software NIS-Elements.

The terminology for larval stages follows Chervy [21] and Palm [10]. The description of tegument surface and microtriches appearance by SEM analysis follows Chervy [22].

### 2.2. Scanning Electron Microscopy (SEM)

For SEM, specimens were fixed in 2.5% glutaraldehyde (0.1 M sodium cacodylate buffer, pH 7.4), washed in the same buffer and post-fixed in buffered 1% osmium tetroxide, dehydrated through a graded ethanol series, critical point dried and sputter coated with platinum. Observations were made using a JEOL JSM 6700F scanning electron microscope operating at 5.0 kV(JEOL, Basiglio, Italy).

### 2.3. Molecular Analysis

For molecular analysis, genomic DNA was extracted from eight larvae using the PureLink^®^ Genomic DNA Kit (Life Technologies, Carlsbad, CA, USA) and following the manufacturer’s instructions. The amplification of the D1-D3 region of 28S rDNA was performed with primers U178_f (5′-GCACCCGCTGAAYTTAAG-3′) and L1642_r (5′-CCAGCGCCATCCATTTTCA-3′) [23]. The thermal cycler program (Tpersonal, Biometra) was 40 cycles of 30 sec at 94 °C, 30 sec at 52 °C and 2 min at 72 °C, preceded by a denaturation step at 94 °C for 2 min and followed by an extended elongation step at 72 °C for 10 min. The PCR products were electrophoresed on 1% agarose gel stained with SYBR^®^ Safe DNA Gel Stain (Thermo Fisher Scientific, Carlsbad, CA, USA) in 0.5X TBE. Amplicons were purified by NucleoSpin Gel and PCR Cleanup (Mackerey-Nagel, Düren, Germany, CA, USA) and sequenced with the internal primers 900F (5′-CCGTCTTGAAACACGGACCAAG-3′) and EDC2 (5′-CCTTGGTCCGTGTTTCAAGACGGG-3′) of Lockyer et al. [23], with an ABI 3730 DNA analyzer (StarSEQ, Mainz, Germany). The DNA trace files were assembled with ContigExpress (VectorNTI Advance 11 software, Invitrogen, Carlsbad, California) and the consensus sequences were compared with previously published data by BLAST tools (https://blast.ncbi.nlm.nih.gov/Blast.cgi). Multiple sequences alignments were constructed using BioEdit 7.2.5 [24]. Pairwise distance, using a Kimura 2-parameter model and maximum likelihood (ML) tree (GTR + G + I, bootstrap of 1000 replicates) were obtained by MEGA version X [25]. The sequences generated in this study were deposited in GenBank under the accession numbers MN660283-89 (*Acanthobothrium* sp.) and MN660290 (*Anthobothrium* sp.).

## 3. Results

Cestodes larvae were recovered in 44% of examined octopuses (14 specimens), with intensity values ranging from 1 to 3. Particularly, two larval forms were found in the digestive tract and were assigned to two distinct orders on the basis of their general morphology: 19 Onchoproteocephalidea larvae were recovered from the caecum and intestine of 9 octopuses (prevalence 28%, mean intensity 2.11) and 5 “Tetraphyllidea” larvae were found in the caecum and intestine of 4 octopuses (prevalence 13%, mean intensity 1.25). Furthermore, a single specimen (prevalence 3%), assigned to the Trypanorhyncha, was recovered from the mantle cavity of a single octopus, adhering to the external wall of the crop.

Coinfection of different larval forms was not observed. All the collected larvae were alive, moving vigorously by rapid contractions and extensions of their body. Descriptions and morphometric features in micrometers (µm; mean followed by the range in parentheses) are provided below.

### 3.1. Morphological Description

#### 3.1.1. *Acanthobothrium* sp. (Onchoproteocephalidea: Onchobothriidae) plerocercoids

**Figure 1:**
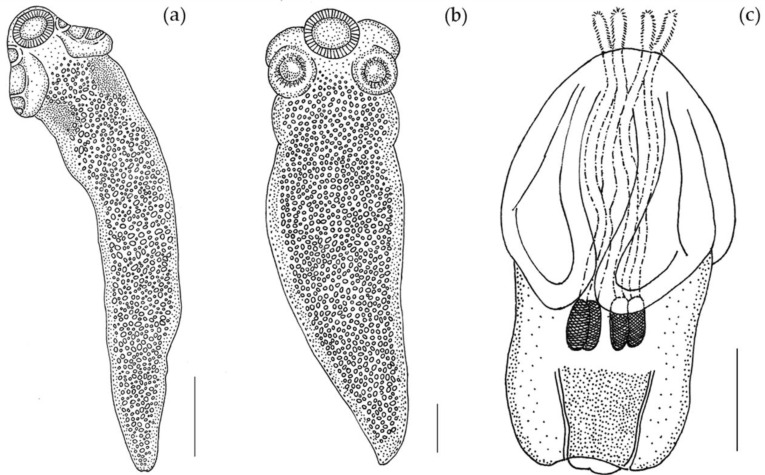
Line drawing of larvae from *O. vulgaris*. (**a**) larva of *Acanthobothrium* (Scale bar: 300 µm); (**b**) larva of *Anthobothrium* (Scale bar: 140 µm); (**c**) larva of *Nybelinia* (Scale bar: 500 µm).

**Figure 2:**
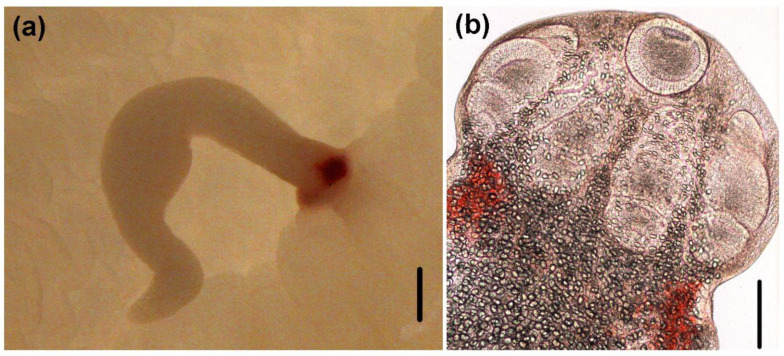
Light micrographs of larvae of *Acanthobothrium*. (**a**) attached to the intestinal lumen of *O. vulgaris* (Scale bar: 500 µm; (**b**) scolex (Scale bar: 100 µm).

**Figure 3:**
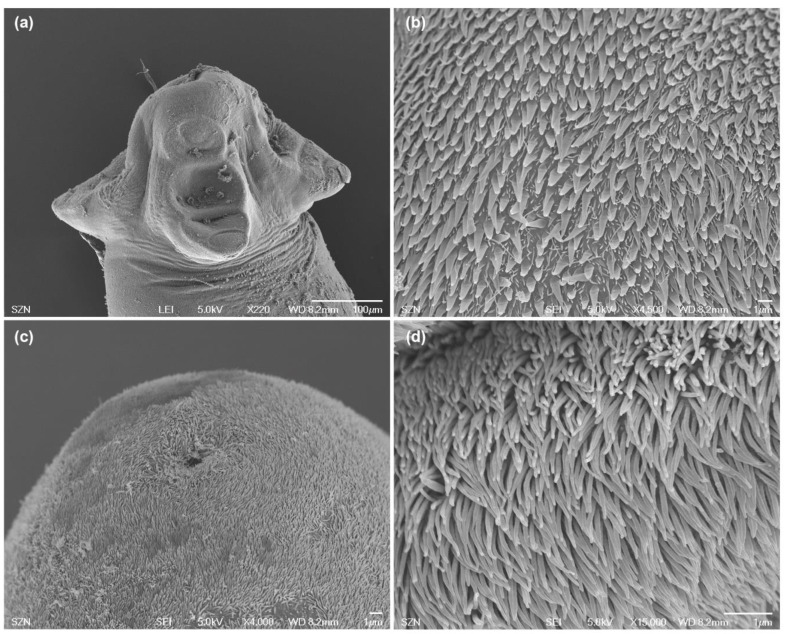
Scanning electron micrographs of larvae of *Acanthobothrium* from *O. vulgaris*. (**a**) scolex, lateral view; (**b**) detail of bothridial surface with capilliform filitriches and gladiate spinitriches; (**c**) caudal end, sub-apical view; (**d**) detail of surface of the caudal end with capilliform filitriches.

(Measurements based on six specimens). Body elongated, 2053.5 (1146.7–3271) total length × 384.4 (214–652) maximum width, tapering posteriorly (Figure 1a). Posterior to the scolex, a pair of bright red pigmented spots are visible in living specimens (Figure 2a,b). Scolex 351.6 (227–484) long × 445.8 (307.3–773) wide, with large apical sucker and four elongated acetabula in form of bothridia; apical sucker 127.7 (88–172) long × 145 (109–195) wide; bothridia, 277.8 (183.9–363) long × 134.7 (86–178) wide. Bothridia sessile anteriorly, free posteriorly, divided into apical pad and posterior loculus (Figure 3a). Posterior loculus subdivided into three loculi. Hooks not yet developed. Parasite tegument characterized by acicular filitriches and gladiate spinitriches over bothridial surface and neck region (Figure 3b); capilliform filitriches covering entire larval body including caudal region (Figure 3c,d).

#### 3.1.2. *Anthobothrium* sp. (“Tetraphyllidea”: Clade 2) plerocercoids 

**Figure 4:**
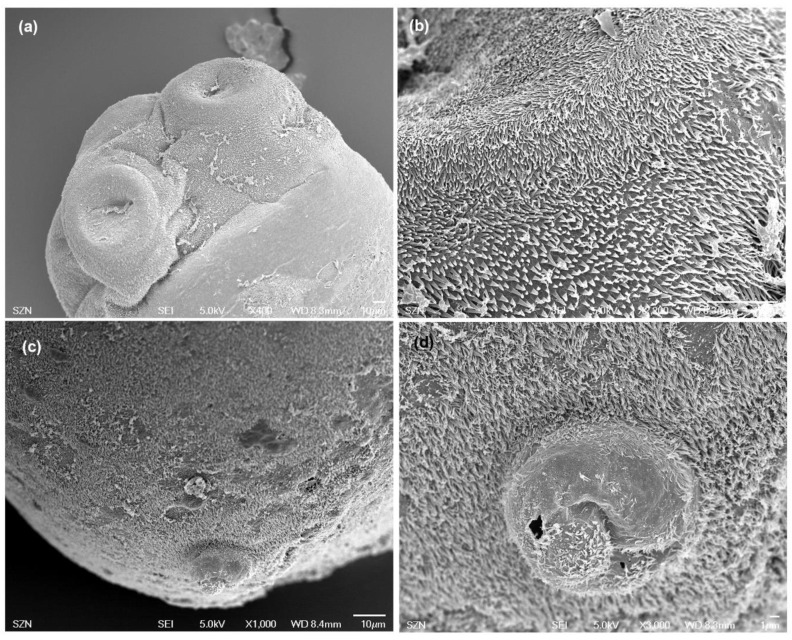
Scanning electron micrographs of larvae of *Anthobothrium*. (**a**) scolex, lateral view; (**b**) detail of surface of acetabula showing capilliform filitriches on distal acetabular surface and gladiate spinitriches on proximal bothridial surfaces; (**c**) caudal end, sub-apical view; (**d**) detail of caudal end.

(Measurements based on two specimens). Body elongated, brownish, 1029 (1007–1051) total length × 344 (315–373) maximum width, tapering posteriorly (Figure 1b). Scolex 196 (181–211) long × 404 (392–416) wide, with apical sucker and four sessile acetabula (Figure 4a). Apical sucker 130 (112–148) long × 110 (102–118) wide, acetabula in the form of suckers 157 (151–163) long × 131 (127–135) wide. Larval body and scolex covered with capilliform filitriches (Figure 4b–d).

#### 3.1.3. *Nybelinia* sp. (Trypanorhyncha: Tentaculariidae) plerocercoid

**Figure 5:**
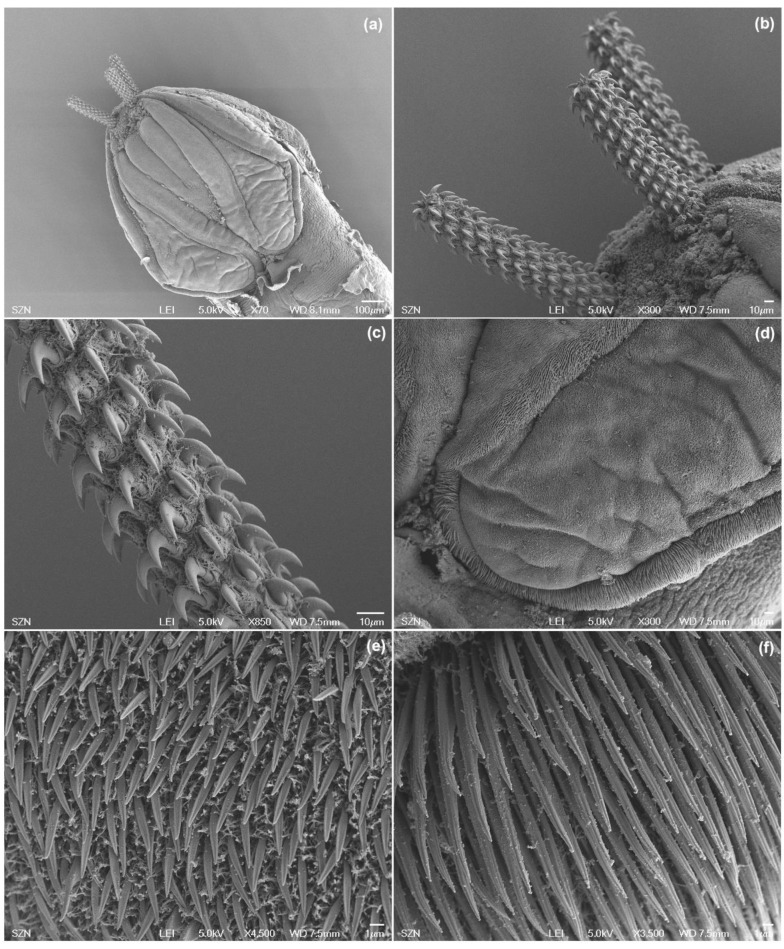
Scanning electron micrographs of larvae of *Nybelinia*. (**a**) scolex with partially everted tentacles; (**b**) detail of the tentacles; (**c**) metabasal armature of tentacle with uncinate hooks arranged in diagonal spiral rows; (**d**) detail of distal bothrial surface; (**e**) detail of capilliform filitriches and digitiform spinitriches on distal bothrial surface; (**f**) detail of bothrial border with longer digitiform spinitriches.

(Measurements based on a single specimen). Body pyriform (Figure 1c), 2269 μm total length × 1000 μm maximum width. Scolex 1220 μm wide, with two bothria; bothria 1370 μm long × 806 μm wide (Figure 5a). Prebulbar organs absent, tentacular bulbs 484 μm long. Appendix 622 μm long. Velum 556 μm long. Presence of four slender cylindrical tentacles 237 μm long (everted part) × 39 μm maximum width, slightly attenuated at the ends. Armature homeoacanthous, homeomorphous (Figure 5b,c). Metabasal hooks uncinate 15 μm long × 12 μm wide, arranged in diagonal spiral rows. Surface of bothria (Figure 5d) covered with filitriches and lineate spinitriches (Figure 5e), bothrial border covered with longer lineate spinitriches (Figure 5f).

### 3.2. Molecular Results

**Figure 6:**
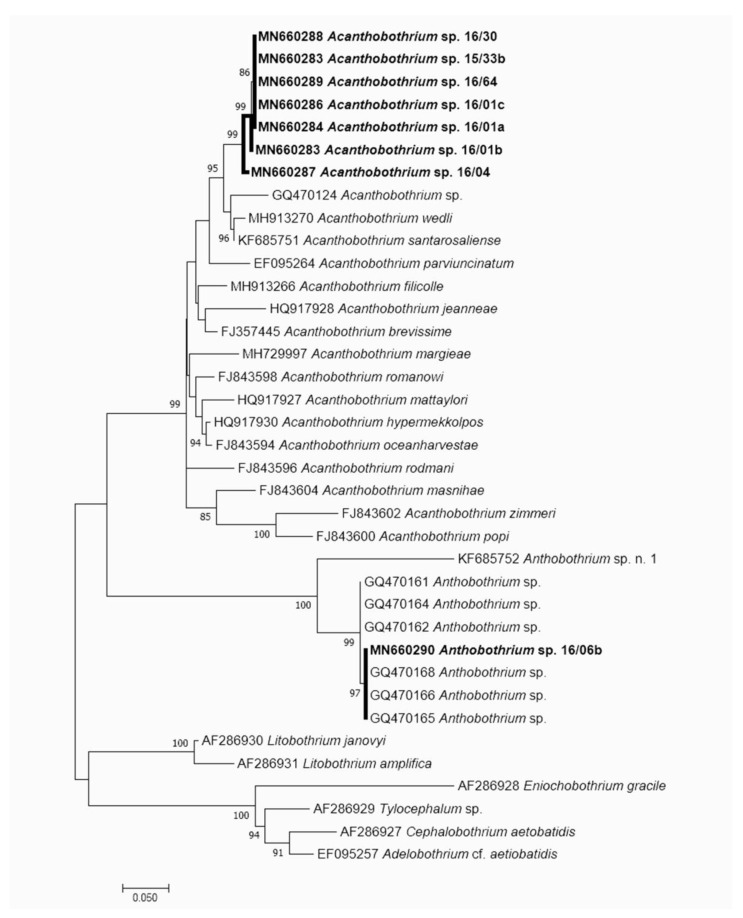
Maximum Likelihood tree (GTR + G + I) showing the relationship with *Acanthobothrium* sp. and *Anthobothrium* sp. described in the present research. The tree is drawn to scale, with branch lengths measured in the number of substitutions per site. The details of the sequences used to build the maximum likelihood (ML) tree are reported in the Supplementary Table S1.

The 28S rDNA of 8 specimens, 7 *Acanthobothrium* sp. and 1 *Anthobothrium* sp. were successfully sequenced and were 1763–1799 bp in length, with a mean divergence 0–0.12% among them. The BLAST analysis of the 7 specimens identified morphologically as *Acanthobothrium* sp. indicated the highest identity (98%) with *Acanthobothrium santarosaliense* Caira & Zahner, 2001 (KF685751) adults infecting the Mexican hornshark *Heterodontus mexicanus* Taylor & Castro-Aguirre, 1972 fished off Santa Rosalia (Gulf of California) and *A*. *wedli* Robinson, 1959 (MH913270) adults infecting the rough skate *Zearaja nasuta* (Müller & Henle, 1841) off New Zealand, while the single *Anthobothrium* sp. analyzed was 100% (69% coverage) identical with *Anthobothrium* sp. (GQ470168) larva infecting the largehead hairtail *Trichiurus lepturus* Linnaeus, 1758 from the Gulf of Mexico. The multiple alignment of the 7 *Acanthobothrium* showed they were all identical to each other except for one specimen showing some transition and transversion. The Maximum-Likelihood tree (Figure 6) generated aligning our sequences with species of *Acanthobothrium* spp. and *Anthobothrium* spp. 28S rDNA retrieved from GenBank showed our *Acanthobothrium* specimens forming a monophyletic group separated but closely related and well supported with the species *A. santarosaliense, A. wedli* and *Acanthobothrium* sp. *Anthobothrium* from the present study is included in the cluster of the *Anthobothrium* sp. (mean distance 0–0.07%).

## 4. Discussion

Understanding the diversity and life cycle of elasmobranch tapeworms is often problematic due to difficulties in the morphological identification of larval forms [9] and in performing experimental life-cycle studies, insufficient information available in the literature and the frequent reporting of currently unaccepted larval names (e.g., *Scolex pleuronectis*) in the intermediate hosts.

For several orders of elasmobranch tapeworms (e.g., “tetraphyllideans”), the identification of larvae is particularly troublesome, since their scoleces remain less differentiated until they develop in the definitive host, therefore the use of molecular data is often required in order to clarify their taxonomy [8].

In a study aimed at generating a molecular sequence library for a wide range of larval cestodes, in order to elucidate morphological features useful for their identification [8], *Acanthobothrium* and *Anthobothrium* larvae infecting teleosts from the Gulf of Mexico have been characterized with morphological and morphometric analyses and through amplification and sequencing of partial 28S rDNA gene. Their size range and general morphology are in accordance with our description of larval *Acanthobothrium* and *Anthobothrium* from Mediterranean *O. vulgaris*.

Plerocercoids tentatively assigned to the genus *Acanthobothrium* based on the morphology of the bothridia were recovered from the intestine of *O. vulgaris* collected in Roscoff (Northeastern Atlantic, France) [26]. Similar larvae, reported as *Scolex polymorphus*, were found infecting the digestive tract of *Sepia officinalis* Linnaeus, 1758 and *O. vulgaris* collected in the Bay of Arcachon (Northeastern Atlantic, France) [27]. Larvae described by Dollfus [26,27] are similar to the larvae of *Acanthobothrium* described in the present study with respect to their general morphology and the presence of bright red regions in the anterior part of the body, posteriorly to the scolex. However, their bothridia differ in the number of septa dividing the loculus posterior to the accessory sucker: bothridia of larvae described by Dollfus are provided with an anterior accessory sucker and a posterior loculus divided in two parts by one transversal septum, while in our *Acanthobothrium* larvae the posterior loculus is divided in three parts by two transversal septa. In larvae of *Acanthobothrium* identified with molecular methods the number of visible septa in the posterior loculus of bothridia can vary from one to two [8]; based on this evidence we speculate that our *Acanthobothrium* larvae and those described by Dollfus [26,27] in *O. vulgaris* from northeastern Atlantic may belong to the same species of *Acanthobothrium*. Possible routes of infection with *Acanthobothrium* in *O. vulgaris* may be represented by bivalve and gastropod mollusks and decapod crustaceans, that are part of the diet of this octopus species and in which different *Acanthobothrium* spp. have been repeatedly reported [28,29,30].

With 201 *Acanthobothrium* species described so far [31], this genus is the most speciose in the order Onchoproteocephalidea and among tapeworms parasitizing elasmobranchs [32] and includes a majority of species that parasitize batoids and some that parasitize sharks [31,33]. Unfortunately, sequence data are currently available for only 16 *Acanthobothrium* species, therefore the identification to the species level of larvae belonging to this genus remains problematic. Our samples apparently belong either to not yet molecularly characterized species of *Acanthobothrium* or to a new species.

Several *Acanthobothrium* species have been reported in elasmobranchs from the Mediterranean Sea [34,35,36]. Particularly, for the Tyrrhenian Sea most of the records are quite old—*A. benedeni* Lönnberg, 1889 in the pelagic stingray *Pteroplatytrygon violacea* (Bonaparte, 1832); *A. coronatum* (Rudolphi, 1819) Blanchard, 1848 in the dogfish *Scyliorhinus stellaris* (Linnaeus, 1758) and *S. canicula* (Linnaeus, 1758), in the common smooth-hound *Mustelus mustelus* (Linnaeus, 1758), in the picked dogfish *Squalus acanthias* Linnaeus, 1758, the angelshark *Squatina squatina* (Linnaeus, 1758) and the common torpedo *Torpedo torpedo* (Linnaeus, 1758); *A. crassicolle* Wedl, 1855 in an unidentified species of *Raja* Linnaeus, 1758; *A. filicolle* (Zschokke, 1887) Yamaguti, 1959 in *P. violacea*, in the Marbled electric ray *Torpedo marmorata* Risso, 1810 and in *T. torpedo*; *A. musculosum* (Baer, 1948) Yamaguti, 1959 in *P. violacea* and *A. zschokkei* Baer, 1948 in *T. torpedo*.

The genus *Anthobothrium* is currently classified in the non-monophyletic order “Tetraphyllidea” and has not been assigned to a family; the most recent classification scheme assigns this genus to the Clade 2 of the “Tetraphyllidea” [33]. The taxonomy of the “Tetraphyllidea” have more recently been revised in the light of new molecular data, with the creation of new eucestode orders—namely Rhinebothriidea [37], Onchoproteocephalidea and Phyllobothriidea [38]—while other species remain in the non-monophyletic “Tetraphyllidea” until additional molecular data become available [33]. With respect to previous work carried out in cephalopods, the description of our *Anthobothrium* sp. larva is consistent with the description of *Scolex* sp. infecting *Loligo vulgaris* Lamarck, 1798 from Brest, France [26] and from the Bay of Arcachon [27]. Nevertheless, morphological data alone do not allow to assign these larval stages to a genus, since larvae with unilocular bothridia could be tentatively assigned to genera belonging to different cestode orders [8,39]. To the authors’ knowledge, no confirmed report of *Anthobothrium* in cephalopod hosts was so far available in the literature.

Similarly to *Acanthobothrium*, the identification of our *Anthobothrium* larvae to the species level is hampered by the scarcity of molecular information: this genus currently includes 16 valid species [33], however sequence data of only two species (*A. laciniatum*, *A. caseyi*) are available.

In the Tyrrhenian Sea, two species of *Anthobothrium* are reported in different elasmobranchs: *A. auriculatum* (Rudolphi, 1819) in *T. torpedo* [40], in *T. marmorata* and in *S. squatina* [41,42,43] and *A. cornucopia* Van Beneden, 1850 in *S. squatina* [43]. The diet of these host species consists of a variety of vertebrate and invertebrate preys, including cephalopods [44,45]. A few additional *Anthobothrium* species are reported from different parts of the Mediterranean Sea (Supplementary Table S1).

Among Trypanorhyncha, the species *Nybelinia lingualis* and *Tentacularia coryphaenae* Bosc, 1802 are the most commonly encountered in cephalopods from Mediterranean and extra-Mediterranean areas. Particularly, *N. lingualis* has been reported in *O. vulgaris* from the Mediterranean Sea and English Channel [1,18,19,20,46,47,48]. Scolex features, body size and general morphology of the *Nybelinia* specimen characterized in the present study are consistent with the description of *N. lingualis* reported in *O. vulgaris* and *Todaropsis eblanae* (Ball, 1841) from NE Atlantic [1,27,49]; particularly, detailed observations by SEM allowed to observe the form and arrangement of hooks over the tentacles and the appearance and distribution of microtriches, adding further detailed information on the microthrix pattern over bothrial surface. Nevertheless, due to the scarcity of material available (only one specimen) and to the lack of molecular data it is not possible to confirm the identity of the collected larva to the species level.

## 5. Conclusions

Our results suggest a possible important role of *O. vulgaris* in the transmission of elasmobranch tapeworms, particularly Onchoproteocephalideans. With respect to tripanorhynchs, this role is arguably less important as compared to ommastrephid squids, where prevalence can be higher than 90% and more than 200 larvae can be found in a single squid [14].

Furthermore, the present work provides, for the first time, detailed morphological (SEM) and molecular support to confirm the occurrence of *Anthobothrium* sp. in cephalopod hosts and the presence of *Acanthobothrium* sp. infection in *O. vulgaris* from the Mediterranean Sea.

## Supplementary Materials

The following are available online at https://www.mdpi.com/2076-2615/10/11/2038/s1, Table S1: List of the sequences used for build the ML tree, with details of the developmental stage, host, locality and specific references.
